# Strengthening the role of community health assistants in delivering primary health care: the case of maternal health services in Zambia

**DOI:** 10.1186/s12875-025-02829-7

**Published:** 2025-05-10

**Authors:** Olatubosun Akinola, Nelia Banda, Adam Silumbwe, Chama Mulubwa, Malizgani Paul Chavula, Hilda Shakwelele, Sylvia Chila, Joseph Mumba Zulu

**Affiliations:** 1 Clinton Health Access Initiative, Box 51071, Lusaka, Zambia; 2 Department of Health Policy and Management, School of Public Health, University of Zambia, PO Box 50110, Lusaka, Zambia; 3 Centre for Community Health Systems and Implementation Research, Lusaka, Zambia; 4 Department of Epidemiology and Global Health, Umea University, Umeå, Sweden; 5 Department of Health Promotion and Education, School of Public Health, University of Zambia, PO Box 50110, Lusaka, Zambia; 6 Ministry of Health, Ndeke House, P.O Box 30205, Lusaka, Zambia

**Keywords:** Community health systems, Community health assistants, Maternal and child health services, Zambia

## Abstract

**Introduction:**

Many low-and middle-income countries, including Zambia experience a huge deficit of human resource for health, which affects the delivery of primary health care services such as maternal and child health (MCH), nutrition, HIV and gender-based services. The Clinton Health Access Initiative in collaboration with the Zambian Ministry of Health implemented a community health systems (CHS) strengthening project to enhance the capacity of community health assistants (CHA) to provide MCH services from 2019 to 2021. The project activities included capacity building in supervision, provision of financial incentives and logistics. This study explores how these interventions strengthened the role of the CHAs in delivering MCH services.

**Methodology:**

This was a qualitative study consisting of 189 KIIs and IDIs as well as 20 FGDs conducted in all the 10 provinces of Zambia with the CHAs, and their supervisors, health workers, neighbourhood health committees and community members. Data were analysed using thematic analysis.

**Results:**

The CHS strengthening interventions including provision of training manuals, streamlined recruitment and deployment policies, capacity building of CHA supervisors, provision of transport and monthly remuneration contributed to improved delivery and acceptability of MCH services. Further, the leveraging of community networks, linkages and partnerships when delivering these services, including the traditional and religious leaders contributed to improved coverage and acceptability of MCH services. Meanwhile, health systems barriers such as limited supplies in some health facilities, shortage of health workers, persistent transportation challenges and failure to fully abide by the CHA recruitment and selection criteria affected delivery and acceptability of MCH services.

**Conclusion:**

This study builds on existing evidence on the importance of building a stronger community–based primary health care to effectively address maternal and child health related issues. We emphasize the need to integrate strategies such as provision of training manuals, enhanced recruitment and deployment policies, capacity building of supervisors, provision of transport and remuneration within the CHA program to enhance the provision and acceptability of health services.

**Supplementary Information:**

The online version contains supplementary material available at 10.1186/s12875-025-02829-7.

## Background

Community health workers (CHWs) have made significant contributions to improving health of underserved communities in most low- and middle-income countries (LMICs) [1–3]. For example, they have provided community level interventions targeting maternal, newborn, child health, malaria and HIV/AIDS services in remote settings [4, 5]. Some specific instances include during the Ebola virus pandemic when CHWs played a critical role in creating awareness and enhancing acceptability of interventions while supporting the health system to deliver routine care [1, 6–8]. Similarly, at apex of the COVID − 19 pandemic, CHWs contributed towards community health systems (CHSs) strengthening through supporting surveillance, contact tracing and quarantine, maintaining essential health services and linking people to care [7, 8]. Their undeniable contribution to continued service provision to communities especially during health epidemics or pandemics underscores the resilience of community health systems [3, 4].

Although CHWs have greatly contributed to improved community health services, gaps still remain with regard to access to primary health care services such as HIV, nutrition as well as maternal and children services [9, 10]. About one-third of the 34 million births in low-income countries including Zambia still take place at home each year without the presence of a trained health care provider [9]. Further, more than 15 million people – mainly children – are still dying every year in LMICs from readily preventable or treatable conditions. Almost one-half of child deaths are due to undernutrition [11]. About 232 million women of the reproductive age in LMICs have unmet need for modern contraception [12]. Adolescent girls and young women (AGYW) in LMICs still experience several challenges that impact their health and well-being such as HIV, poor sexual and reproductive health (SRH), poor access to family planning, and gender-based violence (GBV) [12].

Evidence suggests that primary health care challenges can be more effectively and readily addressed by building a stronger community–based primary health care (CBPHC) [4, 12–14]. This should be done with the full engagement of community–level workers in planning, implementation and evaluation of HIV, nutrition, GBV, maternal and child health programmes [4, 11]. Countries should prioritize strengthening community health systems in order to accelerate progress towards universal health coverage (UHC) and ending preventable child and maternal deaths by 2030 as required by the Sustainable Development Goals and the World Health Organization, and UNICEF respectively [11]. According to Schneider et al., a CHSs comprises a network of local actors and systems that provide and support health in communities but operates in alignment with formal health systems structures [15].

Lately, there has been increased calls for more research to explore CHWs operations and their contribution to CHSs strengthening [16]. This is particularly important owing to CHWs potential to accelerate attainment of UHC, which aims to provide access to essential services without financial hardship [1], through integrated community-level approaches, collective action and upholding social accountability [17]. Additionally, the complexity associated with implementing interventions in CHSs including CHW programs has contributed to increased need to better understand CHS strengthening initiatives [18]. These CHW and CHS program complexities include underfunding [17], challenges in mobilizing multiple stakeholders, the dynamic nature of community contexts [19], and the extensive fragmentation associated with CHW programs [20]. Moreover, the emergence of the COVID- 19 pandemic and the role that CHWs played in supporting health systems to continue delivering health services has also contributed to increased attention towards CHS strengthening [21].

### The CHA programme in Zambia – policy and program environment

In Zambia, a national CHW strategy was introduced in 2010 with the aim to train 5,000 Community Health Assistants (CHAs) by 2021 who would contribute to alleviating the health workforce gap [22]. The core components of this strategy are standardised recruitment and deployment, training, incentives and supervision of CHAs. As of 2020, there were 3,191 CHAs deployed across Zambia with 2,142 of them on government and international partner payroll, while 1,049 were volunteering. These CHAs were recruited from across all the 10 provinces based on the CHW strategy guidelines. Two CHAs were selected per health facility and to be recruited, one should have completed Grade 12 and must possess a school certificate [22]

The CHAs have been essential in mitigating some of the PHC challenges at community level through awareness creation, generating demand, identifying, and making follow-ups [22]. However, the CHAs have also faced several challenges that impact their capacity to perform assigned functions [16, 23]. The widely documented challenges include inadequate incentives, weak supervision, unclear career progression pathways as well as logistical challenges including limited transport [24, 25]. These challenges affect their contribution towards health outcomes as well as community health systems strengthening [24].

### CHAI support towards strengthening the role of CHAs to deliver primary health care

Under USAID's Community Health Systems Strengthening initiative, the Clinton Health Access Initiative (CHAI) implemented several activities to complement existing government efforts to support CHAs'capacity to deliver PHC services, including MCH. For example, to address financial challenges, CHAI provided monthly salary support to 600 CHAs for 18 months. In addition, to alleviate problems of transport and other essential logistics, CHAI supported the procurement and provision of supplies such as bicycles, bag packs and raincoats to enable CHAs to provide PHC in communities, including MCH-related health education. It also supported 175 CHAs to obtain their Health Professionals Council of Zambia (HPCZ) practice licences and trained CHA supervisors to support and mentor CHAs after deployment.

Although it is widely acknowledged that CHWs are central human resource actors in CHS, there remains several gaps with regards to understanding other systems elements essential for building functional and effective CHSs. This paper documents the essential components for strengthening the role of community health assistants in delivering primary health care in the context of maternal and child health services in Zambia.

## Methodology

### Study context

Zambia has made some good progress in reducing child and maternal mortality – a core aspect of strengthening PHC. The maternal mortality ratio significantly decreased from 729 deaths per 100,000 live births in 2002 to 118 in 2022 [26]. Investments in health the workforce within the community health system has contributed to this progress. Community-based action groups and workers have for instance helped increasing coverage and uptake of maternal and newborn health services in rural districts by providing services closer to the people [27, 28]. Yet, the country still experiences significant challenges as the maternal mortality ratio remains high, especially in rural areas [27]. This is a great concern given that about 60% of Zambians live in rural areas in extreme poverty [19].

### Study design

We employed a qualitative case-study design to explore community health systems interventions for strengthening the role of community health assistants in delivering primary health care in Zambia [29]. The methodology section has been organized in line with key aspects of the criteria for reporting qualitative research (COREQ) guidelines such as description of study setting, selection and recruitment of participants, data collection and analysis [30].

### Conceptual model of community systems strengthening

We adopted the conceptual model of community systems strengthening (CSS) developed and revised by the Global Fund in 2014 as a model for organizing the interview guides and interpreting the results [31]. This framework was chosen because of its systematic approach to CSS, emphasizing core interventions necessary for establishing functional and effective community systems that enable community organizations and diverse actors to contribute to local health outcomes.

**Fig. 1:**
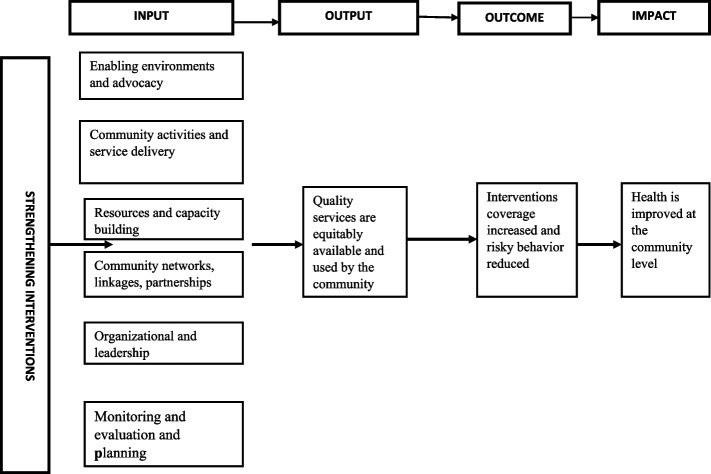
Overview of a strengthened community system. Adapted from The Global Fund 2014 (https://www.theglobalfund.org/media/6428/core_css_framework_en.pdf)

The framework identifies six critical interventions that can catalyse strengthening of health systems. Firstly,* community activities and service delivery* – accessible to all who need them, evidence-informed and based on community assessment of resources and needs. Secondly, *enabling* e*nvironments and advocacy* – including community engagement and advocacy for improving the policy, legal and governance environments affecting the social determinants of health. Thirdly, *community networks, linkages, partnerships, and coordination* – enabling effective activities, service delivery, maximizing resources and impacts, and coordinated, collaborative working relationships. Fourthly, *resources and capacity building* – including human resources with appropriate personal, technical, and organizational capacities, financing (including operational and core funding) and material resources (infrastructure, information and essential medical and other commodities and technologies). Fifthly, *organizational and leadership strengthening* – including management, accountability and leadership for organizations and community system. Lastly, *monitoring and evaluation and planning* – including M&E systems, situation assessment, evidence-building and research, learning, planning and knowledge management (Fig 1).

### Data collection methods

Data were collected between November and December 2020, using semi-structured interviews. These included key informant interviews (KIIs) with health workers, in-depth interviews (IDIs) with the CHAs and the focus group discussions (FGDs) with community members including the neighbourhood health committees (NHC) members. Qualitative data comprised perspectives and experiences regarding access to HIV, nutrition, MNH and GBV services. In addition to exploring delivery of services by the CHAs, the KII also addressed policy related matters. The IDIs only focused on experiences of CHAs in delivering services while the FGDs explored community members’ experience of the services.

Data were collected by a team of six independent researchers trained in data collection at undergraduate or postgraduate level. Researchers who had not worked with CHAs before were engaged to collect data to promote freedom of expression among the CHAs and community members during the interviews. The Principal Investigator (PI), from the University of Zambia, whose PhD study focused on evaluating pilot process of the CHA program, trained and supervised the research assistants. The PIs’ background enabled the study to develop data collection tools that were relevant to the study context and the program.

The KIIs and IDIs were conducted from the office or in private room at the health facility to ensure confidentiality. The FGD were conducted within the community to avoid mobility challenges. The IDIs, KIIs and FGDs interviews were conducted in both English and local languages spoken by the participants. A total of 189 KIIs and IDIs as well as 20 FGDs were conducted. The KIIs and IDIs lasted approximately 30 to 60 min while the FGDs lasted between 60 and 90 min (Tables 1 and 2).

**Table 1:** Individual interviews sample

CHAI-CHA Evaluation	
**Type of interview**	**Total interviews per category (study participants)**
KII with CHA Supervisors at the health facility	57
KII with District Health Staff	33
KII with Province Health Staff	18
KII with CHA Trainers	6
KII with CHA National Coordinator	3
IDIs with CHAs	72
**TOTAL**	189

**Table 2:** Focus group discussion participants

Type of interview	Districts	FGD per district	Number of participants per FGD	Total
FGD with NHC members	10	1	10	100
FGD with community members	10	1	6	60
Total				160

### Selection of participants

Purposive sampling was used to select participants for the study. This sampling process enabled us to sample participants that have worked or interacted closely with CHAs at community, district and national levels. All ten provinces were included in the study. In Each province, one rural district was sampled as CHAs were introduced to deliver services in rural areas. CHAI was operating in six (Northen, Muchinga, Copperbelt, Central, Luapula, and Northern provinces) of the ten selected provinces. Sample selection for community members was guided by key considerations such as community experiences in accessing CHA services or interactions with CHAs.

#### Data analysis

The audio recordings from the interviews were transcribed verbatim. For interviews that took place in local languages, the transcripts were translated into English. This process was conducted by trained research assistants while the co-authors reviewed and cleaned the transcripts to ensure consistency. The transcripts were de-identified to maintain participant confidentiality, meaning that any names or other identifying information were not included in the final transcript.

We followed a thematic analysis approach, which is “a method for identifying, analyzing, and reporting patterns (themes) within data. It minimally organizes and describes a dataset in rich detail and goes further to interpret various aspects of the research topic” [32]. The coding process, conducted by six research assistants, began with reading and re-reading the transcripts to develop the initial codes. These initial codes were then discussed and refined to create the final coding list. The team subsequently proceeded to code all the transcripts individually. The final coded transcripts were shared with all co-authors for review and discussion of the final coding reports. Ultimately, the codes were merged into overarching themes.

The co-authors comprised a team of researchers from the University of Zambia, CHAI, and the Ministry of Health, with expertise in implementation science, anthropology, and community health—particularly in the work of CHWs. The complementary expertise of the researchers enhanced trustworthiness of the data, as well as its analysis and interpretation.

We employed an inductive analysis approach to map themes related to interventions for strengthening the role of CHAs in PHC from the transcripts onto the conceptual model of CSS. This was feasible because we had incorporated sections of the model into the interview guides.

#### Ethical consideration

Approval to undertake the study was sought from the CHAI Scientific Ethics and Research Committee, the Excellence in Research Ethics and Science and the National Health Research Authority. Written, informed consent was obtained prior to commencement of the interviews. Informed consent was sought in a private place so that participants could freely choose whether to participate in the study. Furthermore, participants were informed that they could withdraw from the study or stop the interview at any point when they felt that they did not want to participate and that such a decision would not attract any penalty. COVID- 19 prevention measures such as masking, observing social distance and sanitising were observed. This study adhered to the Declaration of Helsinki.

#### Findings

These findings are grouped according to the broad themes related to inputs in the CSS framework, community activities and service delivery, community networks and coordination, resource sand capacity building, organizational and leadership strengthening, and monitoring, evaluation and planning. The theme on enabling environments has been presented in the background section under heading –the CHA programme in Zambia – policy and program environment.

#### Community activities and service delivery

### The role of CHAs in delivering maternal, neonatal and child health services

For maternal, neonatal and child health, the participants reported that CHAs delivered health education including teaching pregnant women on the importance of nutrition, early prenatal booking, attending antenatal and postnatal services, danger signs in pregnant women, and malaria prevention (i.e., sleeping under a mosquito net). CHAs also played a critical role in educating pregnant women on the importance of delivering from the health facilities and utilizing the health services.

*“They (CHAs) do teach us yes sometimes that if you are pregnant, you are supposed to be coming for antenatal. They teach a lot of things such as taking care of the body and pregnancy, hygiene, cleaning yourself in armpits and private part, budgeting for the baby before giving birth” (224, FGD Community, Itezhi Tezhi District, Central Province).*

With regards to child health services, the CHAs made follow-ups with parents to ensure that mothers took their ill children to the clinic and that those who went for postnatal services were taking proper care of their infants. During these visits mothers were also encouraged to ensure their infants received all the required immunizations, as one participant said:

“…*when the baby is discharged and the mother from the facility they go there to visit them they make sure that they come for postnatal here at the first six days, if they came they have to continue visiting them even at six weeks they have to visit them to encourage them to come to the facility for those vaccines that we give them if they find one who has not come here*” (94, IDI, Female In-Charge, Nakonde District, Northern Province).

In addition to encouraging parents to continue with postnatal services as indicated in the quote above, participants reported that they were aware that CHAs provided child health services through under five services such as weighing, and measuring the length and height of children, vaccinations, and nutrition programmes.

***“****Yes, I know the activities that CHAs provide, and these activities include immunization and some components of malnutrition I think that’s all. Ahh he normally does that; he helps on taking the weight of the baby and checks on card to check the nutrition status of the baby. Yes, is the babies weight improving or it's going down.” (110, IDI, EHT, Chadiza District, Eastern Province).*

### Nutritional services

Aside from providing maternal, neonatal and child health services, the CHAs went further by providing information to promoting uptake of nutrition services including teaching parents and community members how to prepare food and balanced diets to avoid malnutrition. Most participants stated that CHAs conducted home visitation to assess the nutritional status of the households, identifying malnourished children and referring those with severely malnourished to the health facility for management.

*“They give us medicine and the child is also given medicine, they also give us instructions on the different types of food to eat so that both the mother and child are fit and maintain strong bodies.’’ (55, FGD, Luwingu District, Northern Province).*

In addition to the above roles, the CHAs taught community members on sanitation issues like constructing toilets and washing hands.

*“When I tell them to clean surroundings, make huts where to cook from and have toilets and hand basins near toilets and they do those things then they are supporting me. They also welcome us in their homes when we educate them about different things. Then they are using mosquito nets to prevent malaria, and they have also dug pits”* (227, IDI, Female, Kawambwa District, Luapula Province).

### Sexually transmitted infections and HIV Services

The CHAs disseminated information in communities concerning the use of condoms to prevent the transmission and infection of HIV and sexually transmitted infections as well as the importance of HIV testing and prevention of mother to child transmission. The CHAs would go into the communities to provide voluntary counselling and testing services in people’s homes and help dispense condoms.

*“They teach us how to protect ourselves against it, for example if someone is sick at home, they encourage us that if we want to have sex, we need to use condoms. If someone in the house who is HIV positive gets pregnant, we need to take them to the clinic so that they are taught on how to take care of themselves”* (224, FGD, Itezhi Tezhi District, Central Province).

Furthermore, respondents reported that CHAs conducted monitoring visits to check the health status for people living with HIV. To support adherence to HIV treatment, CHAs distributed ART to those who were unable to collect for themselves at the facility.

“…, *then if we find to say they are HIV positive we refer them at the health facility that’s where they are now supposed to start accessing services of drugs*” (92, IDI, Male CHA, Nakonde District, Northern Province).

### Gender-based violence services

The CHAs provided gender-based violence services to communities including sensitisation of both adults and children on GBV, counselling, helping to report cases and referral to other institutions dealing with cases of GBV like the police and traditional leadership structures.

*“On Gender based violence, we talk to people about the dangers of Gender based violence and the outcomes of fighting. If we find that there is a case of GBV either towards a man or woman, we report to one stop centre at Nakonde urban clinic then the issue will be followed up from there. Sometimes Others are treated unfairly so that the issue ends there but that doesn’t end so we report so that the person is disciplined and doesn’t repeat, and we create peace between those people”* (77, IDI, Male CHA, Nakonde District, Muchinga Province).

*“They teach us that if your partner beats you, you can come and report at the facility…what I can say is that we have seen a reduction in such cases after they started teaching us in the community. People are now aware that they can report to the facility and the headman”* (254, IDI, FDG, Kawambwa District, Luapula Province).

#### Resources and capacity building

This resources component includes human resources with appropriate personal, technical and organizational capacities, and material resources like essential infrastructure, medical and other commodities and technologies.

To improve the quality of supervision, the Ministry of Health and CHAI with collaborating partners supported a training programme for CHA supervisors. The training focused on helping CHA supervisors understand the duties, responsibilities, and roles of CHAs as well as how to supervise the CHAs. A total of 456 supervisors were trained. Some CHAs were also helped to obtain their Health Professionals Council Zambia (HPCZ) practicing licenses. CHAI further provided monthly salary support for 18 Months to 600 CHAs.

Participants reported that the training for supervisors enabled them to support CHAs to carry out their duties accordingly. It was reported that the training helped the supervisors to work as good mentors to the CHAs by enhancing their understanding of the roles and duties of CHAs. This situation also improved the working relationships between CHAs and community members.

*“The only training that we conducted I think like once, we take them for practical, we trained some members of staff because you know we are not entirely there with them, so we are trying to orient the members of staff on how to handle CHAs when we deploy them for practical’sessions and that training was only done once”* (261, IDI Tutor, CHA Training School, Central Province).

A few supervisors who had not been trained in supervising CHAs mentioned that they still did not fully understand the roles and skills of the CHAs. They noted that the lack of knowledge affected their ability to engage and support CHAs in their duties. Some supervisors for example could not fully understand the working schedule for CHAs and their responsibilities. This lack of understanding affected effective supervision as noted in the reflections below.

“*I don’t know what CHAs do. I just saw that a CHA was deployed at this health facility. From the time he came, I never offered any supervision because I am not sure about his level of competence*” (Health facility staff, Luangwa District, Lusaka Province).

Some of the CHAs wished to have better supervision from their line managers. They noted that supervision would help clarify their scope of work and motivate them conduct their duties diligently.

*“I wish I can be supervised more and allowed to spend most of the time in the community. Supervision in the community is poor, so I wish to be supervised more”* [227, IDI, Female, CHA, Kawambwa District, Luapula Province].

Despite the training that CHAI provided in implementing and supervising the CHA, it was reviewed that some of the CHAs were not selected from the community they served, and that the advertisements for the CHA positions were not shared with these communities. Sometimes CHAs were transferred from one health facility to another. However, in a few cases when a CHA was drawn from a different community, the receiving community had reservations in trusting them, as they did not know them well, and consequently some members found it hard to open their homes to CHAs. Language barrier also played a huge role, in instances where a CHA was deployed from another place as it was difficult for them to communicate effectively.

*“It only becomes a challenge when you receive someone who is not from the community as I mentioned earlier because I don’t know the criteria they use when it comes to enrolment so you find that you receive somebody from somewhere else who doesn’t even know the language, so it becomes a challenge for that person to communicate with the people at the facility”* (IDI, In Charge, Kalumbila District).

Another challenge reported by the participants was limited transportation which made it difficult for CHAs to provide PHC services in communities that were far from the health facilities. Participants indicated that only a few health facilities that had been supported by government implementing partners like CHAI had been provided with bicycles to facilitate the provision of services to the communities. Thus, the CHAs walked long distances to provide health services and in some cases, this affected the number of households they visited. Many CHAs requested the need for transportation to adequately provide services as noted in the quote below:

*“The ones that are being supported by CHAI were given bicycles. Each facility, each health post was given a bicycle so those CHAs can use bicycles. Of course, they're not enough. The ideal situation is maybe for us to have for each CHA, a bicycle mode of transport, ---, but each facility has at least one bicycle and one motorbike compared to in the past. So, I would say we are at 60% in terms of transport” (*13, IDI Provincial Health Office, MOH, Muchinga province)

#### Community networks, linkages, partnerships, and coordination

This component includes community level structures and actors that support CHAs in delivering services at community level.

### Coordination among actors

The training that was provided by CHAI on supervision of CHAs also enabled the supervisors to effectively work and coordinate better with community actors. This improved coordination was made possible through facilitating community meetings and allowing CHAs to participate in health facility gatherings with communities. The logistical support by CHAI (including providing supplies like bicycles, bag packs and raincoats) helped CHAs to regularly visit the communities and engage other actors in delivering PHC services. The CHAs engaged and worked with various actors at community level when delivering health services, including the traditional and religious leaders. The CHAs also worked with and received support from the health workers, NHCs, CBVs and local leadership in the community. CHAs also collaborated with other CHWs working on volunteer basis in conducting sensitization and outreach in the community.

*“CHAs work with neighbourhood and community health workers. Then when they have a case in the community, the neighbourhood members they’ll communicate with CHA … the CHA can go and see that patient to see how the patient is and how the child is, so their role is to help this CHA to do their job because most of the neighbourhood, they know what is happening in the community”* (06, IDI, Female, In-charge, Kalumbila District, North Western Province).

The CHAs reported that they were a bridge between the community and the health facility as they had a good relationship with the community members, traditional leaders, and neighbourhood health committees. Since CHAs spent much of their time providing health services in the community, they ensured that relationships with the community and community leaders was good to enable them to do their duties smoothly.

“*The working relationship is just ok. I have the HCC chairperson who is very free with me and helps me with whatever thing I ask. Most of the time we use his personal motor bike to go in deep areas and he does not refuse…so, we have a good health relationship with him and other community members because they know him very well”* (58, IDI Female CHA, Luwingu District, Northern Province).

### Open communication, trust and feeling of comfort with CHA services

The strong community ties between the CHAs and the community, which was partly due to the logical support from CHAI, enhanced open communication and trust. The CHAs indicated that sometimes community members were more comfortable with them than with the health facility service providers. For example, in situations where community members were shy to go for a service such as family planning, they would opt to go through the CHAs. Further, the CHAs mentioned that when they communicated with the community, they listened and took into consideration community views, criticism, and corrections. These relationships and ability to reach out and understand community demands had earned the CHAs trust and respect among community members.

*“I remember there was this time a woman came here, just family planning and then she was like, she found him here and then I was dispensing drugs here. She called me outside, no he is my child, i can’t go there and talk about family planning he will start wondering why I need family planning I even laughed with her that what will happen the time you will get pregnant and maybe in the labour ward you know. So, you find that they are more comfortable talking to me than the person they know”* (182, IDI, Female, CHA, Kalabo District, Western Province).

Community members trusted the CHAs because they kept confidentiality with regards to their health issues, they discussed with them. They stated that they did not hear any of the issues they shared with the CHAs from other community members, which earned them their trust.

*“I trust them, but I am not sure if they trust me. From what I have seen they trust me because they confide in me when they have problems, and they are able to complain when they have issues or when something goes wrong. For example, I told them to dig pits, toilets and make hangers for plates but the duration was short, so they complained and asked for a bit more time to do all the things I told them to do, and they did them within a month”* (227, IDI, Female, CHA, Kawambwa District, Luapula Province).

This feeling of trust was mainly attributed to the relationships that the CHAs had built with the community over time. Some community members stated that they were not afraid of asking the CHAs on anything because they didn’t alienate them, but rather they just corrected them. This made community members satisfied with what the CHAs had taught them. Further, community members explained that CHAs cared about the community members, and they are mature people who encouraged self—expression. The healthcare providers were equally of the view that the CHAs were accepted in the community. They stated that in some instances where the CHAs were not seen, the community members would directly go to the health facility and enquire about them. Further, the health providers indicated sometimes community members would prefer to be attended to by the CHAs.

*‘’We have welcomed them because they are open to us, we are not afraid of them and regardless of what you want to ask, they don’t laugh at us, they just correct us. We are also very satisfied with what they have taught us. They come to our communities and start doing their job. They do not bring any problems, and we accept them in the community the way they come. They care about the community members very much and they are mature people who can understand you regardless of how you say something.’’* (70, IDI NHC FGD, Nakonde District Northern Province).

#### Organizational and leadership strengthening

This organizational and leadership strengthening component discusses management and leadership systems for CHAs including CHA supervision system.

### The difficult in balancing work schedules at community level

Some participants stated that the CHAs did adhere to the established working schedule while other participants indicated that this arrangement was difficult to adhere to due to shortage of staff at the health facilities. The problem related to shortage of trained health workforce was more prominent in rural areas which meant that CHAs were mostly working at the health facility. This prevented the CHAs from delivering health in the community as expected.

*“They deliver but it is just the challenge of inadequate human resources, they have been given that 80% they need to go in the community and 20% it’s here at the health facility, now in situations that the in-charge is not there and the community is big and then the government just brings two or one worker so when they are not there it is a challenge in terms of congestion, that is why a person can fail to meet the 80%” (*82, IDI, FGD, Community Members, Luwingu District, Northern Province).

In a few health facilities with shortage of trained health workers, the participants complained that they had never seen CHAs work in the community. The challenge of CHAs not regularly working in the community had made it difficult for some community members to recall the CHA responsibilities as they did not have the opportunity interact with the CHAs on a regular basis.

*“Even in the community, I have never met them before that’s what I can say so, no wonder I am failing to answer other questions because I had met them and seen their work. It would have been very easy answering the questions but now it is very difficult because I have never met them”* (03, FGD, HCC- NHC, Kalumbila District, Northwestern Province).

### Shortage of drugs/commodities

Another key challenge that affected ability by CHAs to deliver PHC services was shortage of drug and commodities. With commodities vital to their service provision, CHAs and health workers felt helpless when they failed to provide drugs to the community members, even when they were able to determine what the problem was. While the drug shortages seemed to be an overall system challenge, the community health work seemed to suffer more, since most of the facility-based drug needs would be prioritised over the community-based needs. As such community health work suffered a more severe case of drug shortages within the community health system. Also, delays in ordering supplies were noted, while some quantities that were ordered at the health facilities were not fulfilled, leading to shortages of drugs for both the facility and the community.

*“The other one is short supply of RDTs, drugs, equipment, and commodities (…) So usually when we order for the facility, we don’t receive the same quantity ordered as we said that there is not enough supply. So, whatever is given to us, is what is shared between the community and facility”* (253, IDI, Female CHA, Kawambwa District, Luapula Province)

#### Monitoring and evaluation and planning

The last component of the model is about the involvement of CHAs in M&E systems, including collection of community level data.

### Facilitating routine collection and timely reporting of data

It was further suggested that the coming in of the CHAs, as well as logistical support from CHAI, did not only facilitate comprehensive availability of data but also ensured timely reporting since the CHA was required to provide information monthly, using the HIA4 A information system (i.e. form developed to aggregate data collected by the CHAs). However, in some facilities CHA reports were reported to be incomplete due to missing data thereby making it difficult for supervisors to provide quality feedback. Previously, the NHCs were responsible for updating the facilities with information about health messages disseminated, household visits and outreach services. One participant highlighted how routine data collection from the NHCs to the CHAs helped to improve the process as follows:

*“It has worked well because we are getting the information from the direct person who is in contact with the community rather than the way it used to be, whereby, it was entirely by the NHCs who were sometimes were reporting or they didn’t report this month, so we discovered that there were a lot of lapses. But with them they are constantly reporting whatever they do in that month”* (242, KII, Male, Community Focal Point Person, Luapula Province).

## Discussion

The study documented community health systems interventions for strengthening the ability of CHAs to deliver maternal and child health services. This study identified the importance of provision of programmatic services such as training, logistical, and transportation as facilitators in delivery of better PHC including MCH services. These helped in promoting their performance by creating a *supportive and conductive environment* which enabled CHAs to deliver quality services in a standardized manner *(community activities and service delivery*). Further, the Ministry of Health also used manuals to address supervisory inadequacies through providing training to the supervisors (*capacity building, organizational and leadership*). Improved and standardized supervisory and monitoring further enhanced the capacity of CHAs to deliver quality services as supervisors had a better understanding of the CHA roles which helped them to confidently mentor the CHAs. The improved ability by CHAs to deliver services especially in the communities resulted into improved *coverage and adoption* of services. These findings relate to other studies that have shown that training, resource allocation and improved supervision of community health workers can contribute to acceptability of their services by enhancing availability of a variety of quality services in communities [14, 16, 33]. Providing services in the communities has the potential of improving adoption and acceptability of services as this practice reduces distances to health facilities [15]. We thus agree that improved training, deployment and supervision of community-based actors can result into strengthened CHS and subsequently promote uptake of various health services and possibly advance population well-being in attaining universal health coverage [34–36].

Further, community health systems strengthening intervention promoted the delivery and uptake of the services through enhancing collective action. This was achieved through strengthening collaboration between CHAs and *community networks, linkages and partnerships* when delivering health services, including the traditional and religious leaders, NHCs and CBVs. Use of community-based resources supported implementation processes of maternal and child health services by enhancing relevance, trust and legitimacy of the services [37–39]. These community-based resources are vital in strengthening community health systems' ability to deliver services as they are “*mechanisms and processes which enable actors in the CHS to mobilize, collaborate and act collectively on health*” [38, 40]. Importantly, the community-based resources promoted coverage penetration of health services by widening participation and accountability as well as promoting trust in health services [11, 14, 34, 41].

The constant interactions with the community, coupled with trust and open communication during outreach sessions placed the CHAs in a unique position to identify and report health emergencies in the community promptly and thus effectively contributing to informing the process of developing responses to these emergencies or disease outbreaks [11, 33]. We note that these relational or community engagement approaches can support development of appropriate services and delivery systems by promoting shared communication and understanding of contextual challenges regarding the delivery and uptake of the maternal and child health services among different actors [42]. This collaborative approach can trigger better adaptation of maternal and child health services at community level services through promoting local ownership [11].

Meanwhile, several health systems barriers affected the ability by CHAs to deliver services, thus undermining community health systems strengthening processes. Limited supplies and medicines in some health facilities, transportation processes to reach distant places and few trained health workers affected CHA operations in communities [15]. Failure by some stakeholders to follow the selection criteria affected acceptability of the CHAs in the community. The contribution of CHAs towards monitoring and planning processes was affected by incomplete reports due to missing data. Uncovering these barriers at the inception of the programme and constantly reviewing these challenges during the implementation process might help to trigger collective action and sustainable relationships aimed at promoting positive change in programme implementation processes [11, 15].

### Limitations and strengths of the study

Devising specific support that CHAI provided related to MCH to ensure effective operationalization was complex as CHAI provided generic PHC support to CHAs. It was expected that this generic support, which included providing training, transportation facilities and incentives, would assist the CHAs to deliver all PHC services including MNC services. The services would include teaching pregnant women on the importance of good nutrition, early prenatal booking, and attending antenatal and postnatal services as well as conducting immunization outreaches. To address this complexity, the team for data analysis and writing consisted of implementers from the Ministry of Health, CHAI and researchers in public health. By systematically highlighting context-specific interventions this work may provide a basis for analytic generalizations that could yield useful insights on essential components for strengthening the role of CHWs in delivering PHC in other similar settings. In addition, transferability of the results was enhanced by providing a rich description of the study context, and by providing quotations in the text representing a variety of informants.

## Conclusion

The study showed that community health systems interventions which included financial support, capacity building of CHA supervisors as well as provision of logistical support contributed to strengthening CHAs’ ability to deliver PHC services. This increased awareness and demand for PHC was because availability of trained CHAs meant that patients could receive services locally. Further, close collaboration among actors within the community and formal health system created a platform for stakeholders to share their experiences thus enabling co-learning and trust which improved service provision and uptake. Although these positive changes were recorded, there were still some challenges which need further attention such as limited availability of drugs and equipment. In addition, some supervisors were not trained in supervising CHAs. To further strengthen CHA operations and the CHS, it is important that the Government implements the strategies outlined in the National Volunteer Policy which was launched in 2022. One major strategy is increased budget allocation to support standardisation of incentives, training, supervision and operational resources for community-based workers such as materials and transportation.

## Supplementary Information

Supplementary Material 1

Supplementary Material 2

Supplementary Material 3

## Data Availability

All data generated or analysed during this study are included in this published article [and its supplementary information files].

## Abbreviations

CHA: Community health assistants
CHAI: Clinton Health Access Initiative (CHAI)
CHWs: Community health workers
HCC: Health centre committee
NHC: Neighbourhood health committee
LMICs: Low-and middle-income countries
MoH: Ministry of Health
PHC: Primary health care
