# Supplementary material for: Strengthening the role of community health assistants in delivering primary health care: the case of maternal health services in Zambia
Source: BMC Prim Care. 2025 May 10;26:156. doi: 10.1186/s12875-025-02829-7 (PMC12065303; doi:10.1186/s12875-025-02829-7)
Supplement: Supplementary file 1 — Supplementary Material 1 [file 12875_2025_2829_MOESM1_ESM.docx]

**Tool CHAs - CHAs and CHS study**

**Introduction**

- Briefly tell us about yourself?
- When were recruited?
- Which CHA training school did you do your training?
- When did you complete the training?
- When were deployed?
- Are you GRZ or partner funded?

1. Effectiveness of CHAs in increasing utilization to basic health services

- What services do you provide in the community?
  - Probe HIV, Nutrition, MNH, GBV services
- How often do deliver services in the community?
- What services do you provide in the health facility?
  - Probe HIV, Nutrition, MNH, GBV services
- How often do you deliver services in the community in month?
- Have you been to adhere 3 days in a week schedule for delivering services in the community versus health facility?
- If you have not been able to adhere to schedule, why not?
- Between the health facility and community, where do you like working from?
- What are the reasons for the preference?
- Which community actors do you work with in delivering services in the community?
  - How is the work relationship/ support?
    - Probe for what has worked well
    - Probe for challenges?

1. Impact of CHAs on health services

- What has been the importance of services that you provide in the community?
  - Probe HIV, Nutrition, MNH, GBV services
- What has been the importance of services in the health facility?
  - Probe HIV, Nutrition, MNH, GBV services

1. Impact of COVID 19 on service delivery

- What services have you provided in relation to COVID 19
- What are the effects of COVID 19 on service delivery

1. Return on investment in CHA program in the community and health facilities

- What has been the added value of having you in this community/ CHAs?
- Do you think it is important for the Government to continue training CHAs, provide reasons for your answer?

1. Training and service delivery

- What has your comment on the training that you received in relation to service delivery?
- How satisfied are you with the training?
- Does your training to help deliver all services well?
  - If not which services do you comfortably provide?
  - Which services do have limitations in delivering?
  - What additional training is required?
  - Which services do you need added training

1. Support to the CHAs including supervision

- What kind of support do you receive from the community?
- What is the relevance of the support?
- Do you receive any supervision at the health facility?
- Who provides supervision at the health facility?
- How often is the supervision at the facility?
- Do you receive any supervision in the community?
- Who provides supervision at the community?
- How often is the supervision at the community?
- What kind of supervision is provided both in the community and health facility?
- Is the supervision different?
- Has there been changes with supervision over time? If yes, what has changed?
- How would rate the quality of supervision at the health facility?
- How would rate the quality of supervision at the community?
- If there anything you would like to change with supervision? If yes, what should be changed?

1. Community health systems software issues and CHAs

- How is the relationship with the community?
- How is the communication between you and community?
  - Probe for ability to freely complain when not happy with services, and how that happens
- How comfortable are you with the community?
  - Probe for trust, participation etc
  - What issues affect trust between the community and you? ***(Probe for impact on formalisation )***
  - What is power relationship between you and various community actors?
  - Probe for types of gender and accessing services
  - Do both women and men freely access the services? Why do you say so?
- How well have been accepted in the community?
- Should CHAs work in the areas where the come? What are the reasons for your answer?

1. Career progression

- What are you career plans?
- What do you think should be the promotion path for the CHAs? Probe for ***(EHT, PHN, EN or midwife)***
- How

1. Programme learning

- How is reporting done?
- What has worked with well with reporting process?
- What are some of the key lesson learned at a result of this project that can be shared and replicated?

1. Gaps and recommendations

- What challenges do you experience when delivering services?
- What recommendations do you have for improving service delivery?
- What recommendations would you have for improving the overall CHA program
