# Supplementary material for: Strengthening the role of community health assistants in delivering primary health care: the case of maternal health services in Zambia
Source: BMC Prim Care. 2025 May 10;26:156. doi: 10.1186/s12875-025-02829-7 (PMC12065303; doi:10.1186/s12875-025-02829-7)
Supplement: Supplementary file 2 — Supplementary Material 2 [file 12875_2025_2829_MOESM2_ESM.docx]

**FGD for NHCs for CHAs and CHS system study**

**Introduction**

1. Recruitment and deployment

- How were the CHAs recruited?
- What role did the NHC play in recruiting the CHAs?
- What is your perception of the recruitment process?

1. Effectiveness of CHAs in increasing utilization to basic health services

- What services do CHAs provide in the community?
  - Probe HIV, Nutrition, MNH, GBV services
  - Probe for how often CHAs deliver services in the community
- What services do CHAs provide in the health facility?
  - Probe HIV, Nutrition, MNH, GBV services
- Which community actors do CHAs work with in delivering services?
  - How is the work relationship?

1. Beneficiaries’ perspectives of the impact of CHAs on health services

- What has been the importance of services provided by CHAs in the community?
  - Probe HIV, Nutrition, MNH, GBV services
- What has been the importance of services provided by CHAs in the health facility?
  - Probe HIV, Nutrition, MNH, GBV services

1. Return on investment in CHA program in the community and health facilities

- What has been the added value of having CHAs in this community?
- Do you think it is important for the Government to continue training CHAs, provide reasons for your answer?
- How would rate the quality of services provided by CHAs? Why do you say so?

1. Support to the CHA including supervision

- What kind of support has the community provided to the CHAs?
- What is the relevance of the support?
- Who is supposed to supervise CHAs?
- Are you involved in supervising the CHAs? If yes, how do conduct supervision?
- What other things can be done by the NHCs to better support CHAs?

1. Community health systems software issues and CHAs

- How is the relationship between the CHAs and the community?
- How is the communication between you and CHAs?
  - Probe for ability to freely complain when not happy with services, and how that happens
- How comfortable are you with the CHAs?
  - Probe for trust, participation etc
  - What issues affect trust between the community and CHAs? Probe for impact on formalisation
  - What is power relationship between the CHAs and various actors?
  - Probe for types of gender and accessing services
  - Do both women and men freely access the services? Why do you say so?
- How well have the CHAs been accepted in the community?

1. Gaps and recommendations

- What are some of the issues which still need attention regarding the operations of CHAs?
- How can these things be improved?
- Overall, what would you want changed/ improved regarding CHAs
