# Supplementary material for: Strengthening the role of community health assistants in delivering primary health care: the case of maternal health services in Zambia
Source: BMC Prim Care. 2025 May 10;26:156. doi: 10.1186/s12875-025-02829-7 (PMC12065303; doi:10.1186/s12875-025-02829-7)
Supplement: Supplementary file 3 — Supplementary Material 3 [file 12875_2025_2829_MOESM3_ESM.docx]

**Tool for National Ministry of Health –CHA/ CHS system study**

**Introduction**

1. Recruitment

- How is recruitment of CHAs done?
- What role does MoH play in recruiting CHAs?
- Are CHAs still recruited from within rural communities?
- What has worked well with CHAs recruitment?
- What recruitment issues still need attention?

1. Training of CHAs

- How is training of CHAs done?
- What role does MoH play in training CHAs?
- How many CHAs have been trained so far?
- What has worked well with CHAs training?
- What training issues still need attention?

1. Deployment

- How is deployment of CHAs done?
- What role does MoH play in deploying CHAs?
- How many CHAs have been employed by Government?
- How many CHAs are being supported by other partners?
- What has worked well with CHAs deployment?
- How many CHAs are not employed?
- What plans are in place to address problems related to deployment?
- What deployment issues still need attention?

1. Reporting

- What kind of data is reported by CHAs?
- How is reporting done?
- How is this information used?
- What has worked with reporting?
- What challenges are experienced during reporting?

1. Supervision

- How is supervision of CHAs done?
- What role does the MoH play in supervising CHAs?
- What has worked well with CHAs supervising?
- What deployment issues still need attention?

What deployment issues still need attention?

- Who else was involved in recruiting CHAs?

1. Impact of CHAs on health services

- What has been the importance of services provided by CHAs in the community?
- How have CHAs been deployed to communities affected the health outcomes of these communities
- How are the conditions of service affecting the CHAs delivery of service ***(Probe for shortage in HWF, Shortage of drugs, commodities, lack of equipment)***

1. Return on investment in CHA program in the community and health facilities

- How much money is spent to recruit one CHA?
- How much money is spent to train one CHA?
- How much money spent to deploy one CHA?
- How much is the salary of one CHA?
- How cost effective is investing in CHAs compared to other carders?
- What has been the added value of having CHAs in this community?
- Do you think it is important for the Government to continue training CHAs, provide reasons for your answer?
- How would rate the quality of services provided by CHAs? Why do you say so?
  - Probe for skills among the CHAs

1. CHA Career progression

- What career path would you recommend for CHAs who want to progress ***(probe for EHT, Nurse, COG)***
- For CHAs with less than five o levels, what steps would you recommend for career progression
- Are there guidelines set for how a CHA can progress to other careers (***Probe for years of serving, ability to take school leave)***
- Are there any sponsorship opportunities for CHAs who want career progression

1. Sustainability

- Has the ministry set in place plans for transitioning CHAs on partner payroll to GRZ Payroll
- Are there plans for placing the unemployed CHAs and the CHAs in training on GRZ Payroll
- What plans have been put in place for sustaining the CHA program

1. Gaps and recommendations

- What are some of the issues which still need attention regarding the operations of CHAs? (***probe for work relations in the community***)
- How can these things be improved?
- Overall, what would you want changed/ improved regarding CHAs
